# *Citrus bergamia* Risso Elevates Intracellular Ca^**2+**^ in Human Vascular Endothelial Cells due to Release of Ca^**2+**^ from Primary Intracellular Stores

**DOI:** 10.1155/2013/759615

**Published:** 2013-11-21

**Authors:** Purum Kang, Seung Ho Han, Hea Kyung Moon, Jeong-Min Lee, Hyo-Keun Kim, Sun Seek Min, Geun Hee Seol

**Affiliations:** ^1^Department of Basic Nursing Science, School of Nursing, Korea University, 145 Anam-ro, Seongbuk-gu, Seoul 136-701, Republic of Korea; ^2^Department of Physiology and Biophysics, School of Medicine, Eulji University, Youngdu-dong, Jung-gu, Daejeon 301-746, Republic of Korea; ^3^KT&G Research Institute, Daejeon 305-805, Republic of Korea

## Abstract

The purpose of the present study is to examine the effects of essential oil of *Citrus bergamia* Risso (bergamot, BEO) on intracellular Ca^2+^ in human umbilical vein endothelial cells. Fura-2 fluorescence was used to examine changes in intracellular Ca^2+^ concentration [Ca^2+^]_i_ . In the presence of extracellular Ca^2+^, BEO increased [Ca^2+^]_i_ , which was partially inhibited by a nonselective Ca^2+^ channel blocker La^3+^. In Ca^2+^-free extracellular solutions, BEO increased [Ca^2+^]_i_ in a concentration-dependent manner, suggesting that BEO mobilizes intracellular Ca^2+^. BEO-induced [Ca^2+^]_i_ increase was partially inhibited by a Ca^2+^-induced Ca^2+^ release inhibitor dantrolene, a phospholipase C inhibitor U73122, and an inositol 1,4,5-triphosphate (IP_3_)-gated Ca^2+^ channel blocker, 2-aminoethoxydiphenyl borane (2-APB). BEO also increased [Ca^2+^]_i_ in the presence of carbonyl cyanide m-chlorophenylhydrazone, an inhibitor of mitochondrial Ca^2+^ uptake. In addition, store-operated Ca^2+^ entry (SOC) was potentiated by BEO. These results suggest that BEO mobilizes Ca^2+^ from primary intracellular stores via Ca^2+^-induced and IP3-mediated Ca^2+^ release and affect promotion of Ca^2+^ influx, likely via an SOC mechanism.

## 1. Introduction

Bergamot essential oil (BEO) is obtained from bergamot (*Citrus bergamia *Risso), a roughly pear-shaped citrus fruit. BEO is widely used in aromatherapy to alleviate symptoms of stress-induced anxiety, mild mood disorders, and cancer pain [1] and it has anxiolytic [2] and analgesic [1, 3, 4] effects in rodents.

Besides these effects of BEO, bergamottin, isolated from nonvolatile fraction of BEO, significantly decreased the typical electrocardiographic signs of coronary arterial spasm, the force of the contraction and the incidence of cardiac arrhythmias induced by vasopressin in the guinea pig [5]. Given the potential roles of bergamottine in cardiovascular function, it is of interest to know the roles of BEO in Ca^2+^ mobilization in endothelial cells. Vasorelaxant effect of BEO may involve Ca^2+^ mobilization from intracellular stores and/or from the extracellular pool in endothelial cells. Several studies demonstrating the relationship between endothelial cells and smooth muscle relaxation have been reported. Our previous study implicated that a change in cytosolic Ca^2+^ levels during stimulation of endothelial cells was a basic mechanism by which endothelial cells modulate vasomotor activity [6]. Another report indirectly supports this view, demonstrating that the vasorelaxant effect of the essential oil of *Ocimum gratissimum *is partly dependent on the integrity of the vascular endothelium [7].

Up till now, the effects of BEO on [Ca^2+^]_i_ in endothelial cells and the mechanisms by which BEO modulates the intracellular Ca^2+^ concentration ([Ca^2+^]_i_) are not revealed. In the present study, we investigate the intracellular Ca^2+^-regulating properties of BEO in endothelial cells and present evidence that BEO increases [Ca^2+^]_i_ via release from intracellular Ca^2+^ stores and through store-operated Ca^2+^ entry (SOC).

## 2. Materials and Methods

### 2.1. Human Vascular Endothelial Cell Culture

A human endothelium-derived cell line EA.hy926 was purchased from the American Type Culture Collection (Manassas, VA, USA) [8]. EA cells were grown in Dulbecco's Modified Eagle Medium (DMEM) containing 20% fetal bovine serum (FBS) and 10% hypoxanthine-aminopterin-thymidine 50 supplement (Life Technologies). Cell cultures were maintained at 37°C in a fully humidified 95% air 5% CO_2_ atmosphere. The media were removed and replaced with fresh medium three times in a week. The cells were detached by exposure to trypsin, reseeded on gelatin-coated coverslips, and maintained in culture for 2 to 4 days before use. Measurements were performed on subconfluent cells.

### 2.2. Cell Viability Assays

The thiazolyl blue tetrazolium bromide (MTT) assay was used to determine the effects of BEO on cell viability. EA cells were cultured using DMEM with 100% (v/v) FBS (Gibco Invitrogen), 100 units/mL penicillin, 100 *μ*g/mL streptomycin (Gibco Invitrogen), and MEM nonessential amino acids (Invitrogen) in 96-well plates (Nunclon, Denmark) for 48 h until 80–90% confluence has been reached. To evaluate the effect of BEO, cells were for 15 min with varying concentrations (0.001, 0.005, 0.01, 0.05, or 0.1% [v/v in DMSO]) of the BEO and 0.25% DMSO. Then cells were washed with fresh PBS and replaced with serum-free media. In addition, cells were loaded with 10 *μ*L MTT (5 mg/mL) and incubated at 37°C for 3 h. MTT solution was removed and replaced with 100 *μ*L DMSO in a dark place for 2 h. The change in color was read at 540 nm using a plate reader.

### 2.3. Ca^2+^ Measurements

Cells were loaded with fura-2 AM, and [Ca^2+^]_i_ was measured using a microfluorometer system consisting of an inverted microscope (IX71, Olympus, Japan) and a PTI Filter Scan power illuminator system (Photon Technology International). Fura-2 AM (2 **μ**M) was added to the bath and the cells were incubated for 25 min at 37°C. The cells were illuminated alternatively at wavelengths of 340 and 380 nm through a chopper wheel (frequency = 50 Hz). Fluorescence was measured at 510 nm, and autofluorescence was subtracted from the signals obtained. The Ca^2+^-free concentration was calculated from the ratio of the fluorescence signals emitted at each excitation wavelength. The calibration procedure was identical to that described previously [9]. 

### 2.4. Solutions and Chemicals

The external solution contained the following (in mM): 150 NaCl, 6 KCl, 1.5 CaCl_2_, 1 MgCl_2_, 10 HEPES, and 10 glucose; pH was adjusted to 7.4 with NaOH. Ca^2+^-free solution contained 5 mM EGTA in place of Ca^2+^. The osmolarity of this solution, as measured with a vapor pressure osmometer (FISKE, USA), was 320 ± 5 mOsm. ATP, 2-aminoethoxydiphenyl borane (2-APB), 2,5-di-t-butyl-1,4-benzohydroquinone (BHQ), carbonyl cyanide m-chlorophenylhydrazone (CCCP), dantrolene, U73122, and MTT were purchased from Sigma; Fura-2 AM was obtained from molecular probes. BHQ, U73122, and essential oil (0.001%, 0.005%, 0.01%, 0.05%, 0.1%, or 0.2% v/v) were applied from a stock solution in dimethyl sulfoxide (DMSO). Final concentration of DMSO was less than 1%. 2-APB was applied from a stock solution in ethanol. The final concentrations of DMSO and ethanol were less than 0.05%. BEO (batch no. 110824) was purchased from Aromarant Co. Ltd., Röttingen, Germany, and came from locally cultivated plants in Italy. 

### 2.5. Statistical Analysis

Pooled data are presented as means ± SEMs and significant differences were determined using paired *t*-test or ANOVA followed by Scheffe's post-hoc analysis. A value of *P* < .05 was considered significant.

## 3. Results

### 3.1. Cell Viability

**Figure 1:**
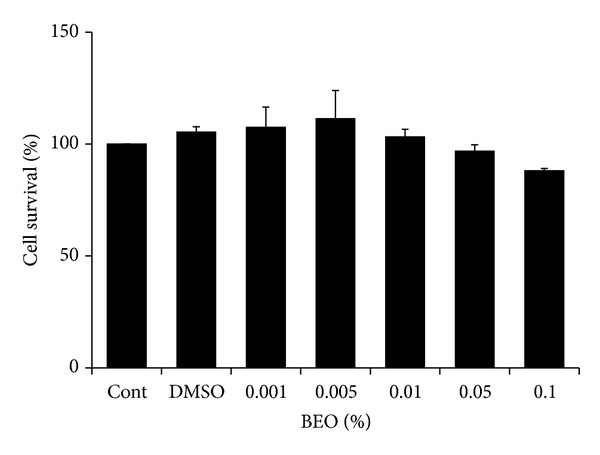
The cell survival percentage measured using MTT assay after 15 min posttreatment of bergamot essential oil, one-way ANOVA followed by Scheffe's post hoc test (*n* = 4).

The MTT assay was used to determine explored effect of varying concentrations of BEO in EA cells. Each cell was treated with media (control), DMSO (vehicle, 0.25% [v/v]), or BEO (0.001%, 0.005%, 0.01%, 0.05%, or 0.1% [v/v in DMSO]) for 15 min (Figure 1). Differences between groups were analyzed using the ANOVA followed by Scheffe's post-hoc analysis. There was no significant effect to the percentage of viable cells at all concentrations of BEO in EA cells (*F*(6.27) = 1.541, *P* = .214).

### 3.2. Elevation of [*Ca* ^2+^]_*i*_ by BEO in Human Vascular Endothelial Cells

**Figure 2:**
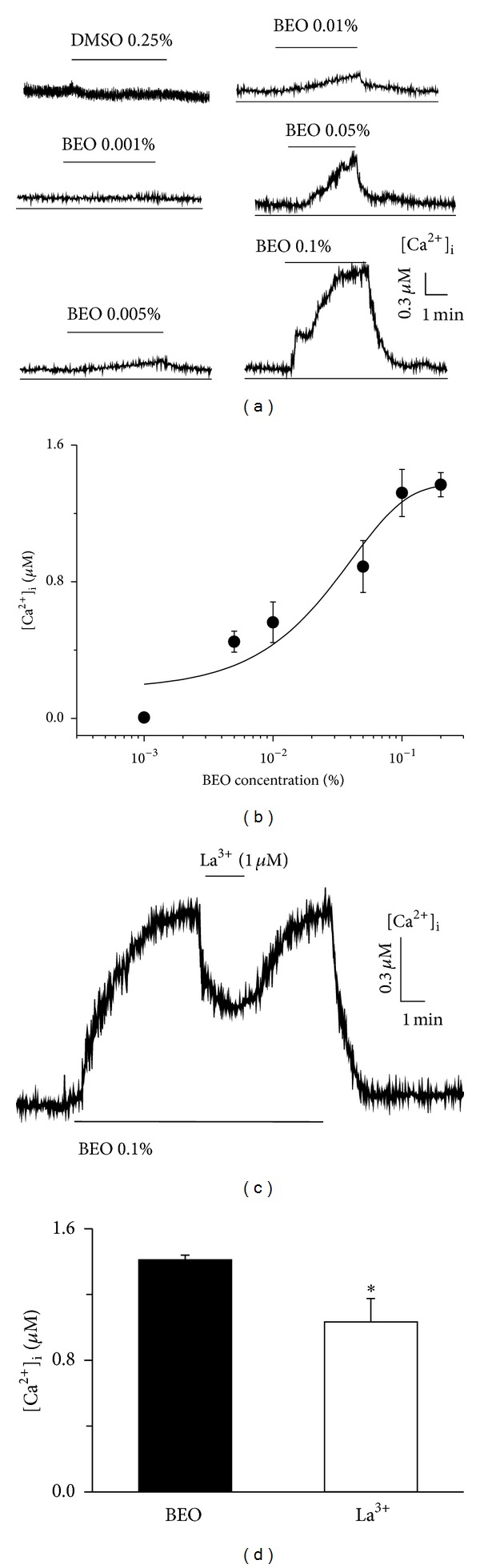
Application of BEO increased [Ca^2+^]_i_ in a concentration-dependent manner (a). Summary data describing the concentration-response relationship for BEO effects on [Ca^2+^]_i_ (b). Data are means ± SEMs. Applications of BEO or drugs are indicated by upper or bottom lines. Effects of BEO on [Ca^2+^]_i_ in human vascular endothelial cells. In the presence of extracellular Ca^2+^, application of BEO (0.1% v/v) induced an increase in [Ca^2+^]_i_ that was significantly inhibited by La^3+^ (1 **μ**M) ((c), (d)). *P < .05 compared to the BEO group; *n* = 10~13 cells/group.

BEO increased [Ca^2+^]_i_ in a concentration-dependent manner in EA cells (Figure 2(a)). The concentration-response relationship for mobilization of Ca^2+^ from intracellular stores by BEO is summarized in Figure 2(b). The concentration of BEO was nonlinearly related to the increase in [Ca^2+^]_i_ as revealed by fitting the Hill equation type dose-response curve. The half maximal increase in [Ca^2+^]_i_ (EC_50_) was obtained at 0.04 ± 0.01%. DMSO (0.25% v/v) itself did not change intracellular Ca^2+^ levels. The cells showed no morphological change after treatment with BEO. Then we investigated whether BEO changed [Ca^2+^]_i_ in the presence of extracellular Ca^2+^ in EA cells. Application of BEO increased [Ca^2+^]_i_ to 1.41 ± 0.14 *μ*M, which was partially and reversibly inhibited by the non-selective Ca^2+^ channel blocker La^3+^ (1 *μ*M). [Ca^2+^]_i_ was reduced to 1.03 ± 0.09 *μ*M by La^3+^ (*P* = .019, *n* = 13, Figures 2(c) and 2(d)), indicating that BEO induces Ca^2+^ influx from extracellular pool and Ca^2+^ release from intracellular stores. 

### 3.3. Ca^2+^ Release from Endoplasmic Reticulum and Mitochondrial Ca^2+^ Stores by BEO

**Figure 3:**
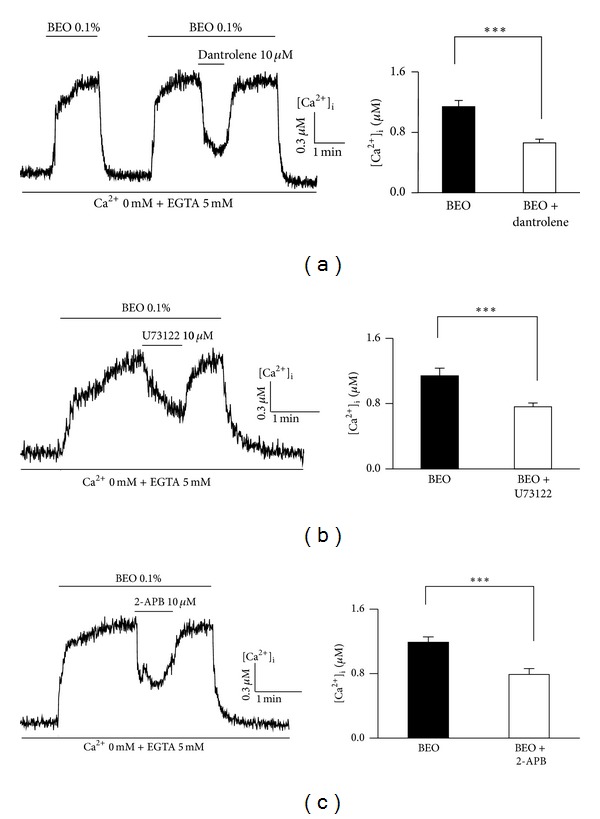
Involvement of CICR and IICR on BEO-induced intracellular Ca^2+^ release. Effects of CICR inhibitor, dantrolene (10 **μ**M) on BEO-induced intracellular Ca^2+^ release. Application of dantrolene partially attenuated increase in [Ca^2+^]_i_ by BEO (the first upper line) (a). Involvement of IICR pathways in BEO-induced intracellular Ca^2+^ release ((b), (c)). Increased [Ca^2+^]_i_ by BEO was significantly inhibited by inhibitors of PLC (U73122, 10 **μ**M) or IP_3_ receptors (2-APB, 10 **μ**M).

We next performed experiments to determine which of the two main dynamic intracellular Ca^2+^ stores, namely, the endoplasmic reticulum (ER) and mitochondria, is affected by BEO in EA cells. Ca^2+^ release from the ER depends on two mechanisms: Ca^2+^-induced Ca^2+^ release (CICR), involving ryanodine receptors, and IP_3_-induced Ca^2+^ release (IICR), involving inositol 1,4,5-triphosphate (IP_3_) receptors [10]. BEO-induced intracellular Ca^2+^ increase was significantly and reversibly inhibited by the CICR inhibitor, dantrolene (*P* < .001, *n* = 10, Figure 3(a)). These data indicate that BEO elevates [Ca^2+^]_i_ in part by the release of Ca^2+^ from intracellular stores via a CICR mechanism. To determine whether BEO releases Ca^2+^ from intracellular Ca^2+^ stores via IICR, we tested the effects of BEO in the presence of U73122, the specific inhibitor of phospholipase C (PLC) [11], to inhibit IP_3_ synthesis, or 2-APB, a membrane-permeable inhibitor of IP_3_-gated ER Ca^2+^ channels [12]. BEO-induced intracellular Ca^2+^ increase was significantly inhibited by both U73122 (*P* < .001, *n* = 10, Figure 3(b)) and 2-APB (*P* < .001, *n* = 15, Figure 3(c)). These data indicate that PLC-mediated synthesis of IP_3_ and IP_3_ binding to IP_3_-gated Ca^2+^ channels in the ER contribute to BEO-induced Ca^2+^ release from intracellular stores.

**Figure 4:**
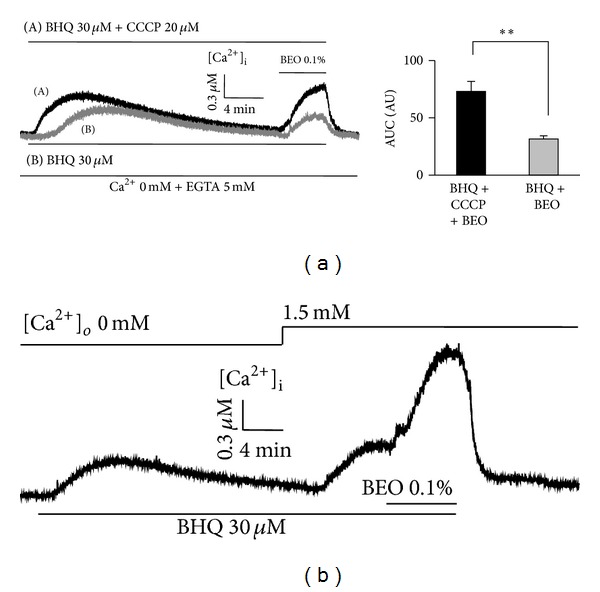
Involvement of mitochondrial Ca^2+^ uptake and SOC pathway on BEO-induced Ca^2+^ release. Inhibition of mitochondrial Ca^2+^ uptake and SR/ER Ca^2+^-ATPase on BEO-induced Ca^2+^ release with CCCP (20 **μ**M) and BHQ (30 **μ**M), respectively (a). Increase in [Ca^2+^]_i_ by subsequent application of BEO in the presence of BHQ and CCCP (black trace a) was higher than that in the presence of BHQ only (gray trace b). Effects of SOC pathway in BEO-induced intracellular Ca^2+^ release. SOC was induced by reintroduction of extracellular Ca^2+^ following SR/ER Ca^2+^-ATPase inhibition with BHQ (b). Intracellular Ca^2+^ levels were further enhanced by subsequent addition of BEO. Data are means ± SEMs. **P < .01; *n* = 15 cells/group. Applications of BEO or drugs are indicated by upper or bottom lines.

A portion of Ca^2+^ released from the ER is taken up by proximate mitochondria, which can also release Ca^2+^ and thereby regulate [Ca^2+^]_i_. To determine whether mitochondria participate in the reuptake of BEO-induced Ca^2+^ release, we examined the effects of BEO on [Ca^2+^]_i_ in a Ca^2+^-free solution in the presence of BHQ (an SR/ER Ca^2+^-ATPase inhibitor) and CCCP (a mitochondrial Ca^2+^ uptake inhibitor). In cells treated with BHQ and/or CCCP, [Ca^2+^]_i_ transiently increased and then decreased slowly to a steady state, suggesting that an SR/ER Ca^2+^-ATPase and mitochondrial Ca^2+^ uptake participate in the regulation of [Ca^2+^]_i_ under basal conditions (Figure 4(a)). Increase in [Ca^2+^]_i_ in EA cells by subsequent application of BEO in the presence of BHQ and CCCP was higher than that in the presence of BHQ only. An area under the curve in each condition was 72.97 ± 8.81 and 31.62 ± 2.75 arbitrary unit, respectively. The difference between two conditions was 41.35 ± 8.12 arbitrary unit, suggesting that mitochondrial Ca^2+^ stores may contribute to the regulation of the BEO-induced increase in [Ca^2+^]_i_. 

Considering the inhibitory effect of La^3+^, noted above, it is suggested that Ca^2+^-entry pathway(s) is (are) activated by BEO. Thus, we next examined whether BEO modulated Ca^2+^ entry via an SOC mechanism. Exposure of EA cells to the BHQ in a Ca^2+^-free solution induced a transient increase in [Ca^2+^]_i_, which then decreased slowly to a steady state (Figure 4(a)). Reapplication of extracellular Ca^2+^ following emptying of intracellular Ca^2+^ stores with BHQ caused an increase in [Ca^2+^]_i_, indicating activation of an SOC mechanism. When BEO was applied after SOC was evoked, SOC was further enhanced, suggesting that BEO also activates Ca^2+^ influx through an SOC pathway (Figure 4(b)). However, using this protocol alone in this experiment does not rule out that other calcium entry pathways may be activated by BEO.

## 4. Discussion 

In the present study, we firstly demonstrate that BEO mobilized Ca^2+^ from extracellular and intracellular sources in endothelial cells. Our present results also suggest that BEO increased intracellular Ca^2+^ level through both mobilization of intracellular Ca^2+^ stores, ER and mitochondria, and promotion of Ca^2+^ influx, via an SOC mechanism. These findings will provide insight into the physiological mechanisms involved in Ca^2+^ regulation in endothelial cells following exposure to BEO.

Some essential oils contain photoactive molecules like furocoumarins. For instance, essential oil of *Citrus bergamia* contains psoralens which bind to DNA under ultraviolet A light exposure producing mono- and biadducts that are cytotoxic and highly mutagenic [13]. Therefore, the results observed in the present study may be results from the effects of photoirritation of BEO. Since, however, intracellular Ca^2+^ level rapidly returned to a baseline level after washing out of BEO and cell viability was normal in doses tested, we think that phototoxic or cytotoxic effect is little on intracellular Ca^2+^ level by BEO. In addition, *in vitro* studies have shown that BEO reduces glutamate receptor-mediated cell death induced by N-methyl-D-aspartate [14]. Nevertheless, further researches are necessary to evaluate phototoxic potential of BEO in endothelial cells.

BEO has been reported to decrease the blood pressure in healthy human and have dilating effect on mouse artery [15, 16]. In the recent study, limonene, one of the major components of BEO (37.26%), increased cytosolic Ca^2+^ concentration by the direct activation of adenosine A_2A_ receptors [17]. In endothelium, an adenosine A_2A_ receptor has an important role in NO release. Adenosine A_2A_ receptor induced NO-dependent vasodilation by intracellular Ca^2+^ increase [18]. Thus, we suggest that the effect of BEO may be attributable to limonene by activation of adenosine A_2A_ receptors.

Contraction and relaxation of smooth muscle are regulated not only by changes in cytoplasmic calcium concentration but also by other important signaling mechanisms, that is, independent of the changes in [Ca^2+^]_i_, known as Ca^2+^ sensitization [19]. Although the increase in [Ca^2+^]_i_ initiates smooth muscle contraction via activating myosin light chain kinase, Ca^2+^ sensitization mediates smooth muscle contraction by modulating myosin light chain phosphatase [20]. The increase in [Ca^2+^]_i_ in endothelial cell plays a role in synthesis and the release of vasoactive compounds such as nitric oxide or prostaglandins [21], thereby altering Ca^2+^ sensitization in smooth muscle cells. It has been reported that linalyl acetate, one of the main components of BEO, induces relaxation of the smooth muscle via partially endothelium-dependent pathway [22]. Further research will be needed to reveal the mechanism by which the calcium releasing actions by BEO in endothelial cells regulate to the vasodilator action.

In conclusion, before BEO can be considered for use in treating vascular-related diseases, further studies are necessary to define the Ca^2+^-elevating pathways enlisted by BEO under pathological conditions.
